# Implementation of the São Paulo Nursing Courses Consortium for the Progress Test: experience report

**DOI:** 10.1590/1980-220X-REEUSP-2023-0347en

**Published:** 2024-06-28

**Authors:** Bruna Moreno Dias, Lúcia Marta Giunta da Silva, Marina de Góes Salvetti, Vanessa Pellegrino Toledo, Silvia Franco da Rocha Tonhom, Marli Teresinha Cassamassimo Duarte, Beatriz Barco Tavares Jontaz Irigoyen, Simone Teresinha Protti-Zanatta, Carmen Silvia Gabriel

**Affiliations:** 1 Universidade de São Paulo, Escola de Enfermagem de Ribeirão Preto, Departamento de Enfermagem Geral e Especializada, Ribeirão Preto, SP, Brazil.; 2 Universidade Federal de São Paulo, Escola Paulista de Enfermagem, Departamento de Administração em Serviços de Saúde e Enfermagem, São Paulo, SP, Brazil.; 3 Universidade de São Paulo, Escola de Enfermagem, Departamento de Enfermagem Médico-Cirúrgica, São Paulo, SP, Brazil.; 4 Universidade Estadual de Campinas, Faculdade de Enfermagem, Campinas, SP, Brazil.; 5 Faculdade de Medicina de Marília, Marília, SP, Brazil.; 6 Universidade Estadual Paulista, Faculdade de Medicina de Botucatu, Departamento de Enfermagem, Botucatu, SP, Brazil.; 7 Faculdade de Medicina de São José do Rio Preto, Departamento de Enfermagem Especializada, São José do Rio Preto, SP, Brazil.; 8 Universidade Federal de São Carlos, Departamento de Enfermagem, São Carlos, SP, Brazil.

**Keywords:** Education, Nursing, Educational Measurement, Academic Performance, Nursing Faculty Practice, Schools, Educación en Enfermería, Evaluación Educacional, Rendimiento Académico, Práctica del Docente de Enfermería, Instituciones Académicas., Educação em Enfermagem, Avaliação Educacional, Desempenho Acadêmico, Prática do Docente de Enfermagem, Instituições Acadêmicas

## Abstract

**Objective:**

To report the experience of implementing the São Paulo Nursing Courses Consortium for the Progress Test.

**Method:**

This is an experience report of the consortium’s work in Progress Test preparation and application for Public Schools of Nursing in São Paulo in 2019, 2021 and 2022, with a descriptive analysis of the work process and the results obtained.

**Results:**

The consortium’s activities are structured into the following stages: planning; theme review; distributing and requesting questions; professor training; question elaboration; question reception; question selection; question validation; student registration; test application; analysis and dissemination of results. A total of 57.3% of enrolled students participated. There was a predominance of questions of medium difficulty and a gradual progression in the level of discrimination of the questions, with, in 2022, 82.5% being considered adequate.

**Final considerations:**

The consortium has allowed the test to be applied interinstitutionally, with greater scope, accuracy, and quality of questions. Through this experience, it is expected to encourage progress testing in undergraduate nursing courses in other contexts.

## RESUMO

**Objetivo:**

Relatar a experiência da implementação do Consórcio dos Cursos de Enfermagem Paulistas para o Teste de Progresso.

**Método:**

Trata-se de relato de experiência da atuação do consórcio na elaboração e aplicação do Teste de Progresso para as Escolas de Enfermagem Públicas Paulistas nos anos de 2019, 2021 e 2022, com análise descritiva do processo de trabalho e dos resultados obtidos.

**Resultados:**

As atividades do consórcio estão estruturadas nas seguintes etapas: planejamento; revisão de temas; distribuição e solicitação de questões; capacitação de docentes; elaboração de questões; recepção de questões; seleção de questões; validação de questões; inscrição de estudantes; aplicação do teste; análise e divulgação de resultados. Participaram 57,3% dos estudantes matriculados. Houve predomínio de questões de média dificuldade e progressão gradual do nível de discriminação das questões, sendo que, em 2022, 82,5% foram consideradas adequadas.

**Considerações finais:**

O consórcio tem permitido a aplicação do teste de forma interinstitucional, com maior abrangência, acurácia e qualidade das questões. Por meio dessa experiência, espera-se fomentar a realização do teste de progresso em cursos de graduação em enfermagem em outros contextos.

## RESUMEN

**Objetivo:**

Relatar la experiencia de implementación del Consorcio de Cursos de Enfermería de São Paulo para la Prueba de Progreso.

**Método:**

Se trata de un relato de experiencia del trabajo del consorcio en la elaboración y aplicación de la Prueba de Progreso para las Escuelas Públicas de Enfermería de São Paulo en los años 2019, 2021 y 2022, con un análisis descriptivo del proceso de trabajo y de los resultados obtenidos.

**Resultados:**

Las actividades del consorcio se estructuran en las siguientes etapas: planificación; revisión de temas; distribución y solicitud de preguntas; capacitación de docentes; elaboración de preguntas; recepción de preguntas; selección de preguntas; validación de preguntas; registro de estudiantes; aplicación de la prueba; análisis y difusión de resultados. Participaron el 57,3% de los estudiantes matriculados. Hubo un predominio de preguntas de dificultad media y una progresión gradual en el nivel de discriminación de las preguntas, considerándose adecuadas en 2022 un 82,5%.

**Consideraciones finales:**

El consorcio ha permitido que la prueba sea aplicada de manera interinstitucional, con mayor alcance, precisión y calidad de preguntas. A través de esta experiencia, se espera fomentar la realización de la prueba de progreso en cursos de pregrado en enfermería en otros contextos.

## INTRODUCTION

The Progress Test (PT) consists of a systematic and longitudinal assessment of student performance. It is used as a tool for cognitive assessment of students’ knowledge acquisition in a continuous and progressive manner. The test results enable teaching-learning process analysis, constituting a feedback tool for students and an academic management instrument, enabling diagnosis and indications of changes in subjects or the curriculum^(1)^.

In medicine, PT implementation as an instrument for assessing undergraduate student performance began in the 1980s in European countries, and is consolidated in courses in countries such as the Netherlands^(2)^, Germany^(3)^ and Saudi Arabia^(4)^. In Brazil, the first experiences of implementing the test in medical schools date back to the end of the 1990s^(5)^.

In healthcare professional education, in addition to its application in medical schools, there are reports of using PT in dental therapy and hygiene and dentistry courses in the United Kingdom^(6)^ and in dentistry courses in Austria^(7)^.

In nursing, the first known initiatives to apply PT are from *Universidade de São Paulo Escola de Enfermagem de Ribeirão Preto* (EERP-USP), *Universidade Federal de São Paulo Escola Paulista de Enfermagem* (EPE-UNIFESP) and *Universidade de Campinas* (UNICAMP) School of Nursing. These isolated experiences and initiatives, although guided by the same premises and objectives, did not allow for benchmarking, or a more comprehensive analysis, as they were structurally distinct and focused on the curricular contents of these schools. As of 2019, the establishment of the São Paulo Nursing Courses Consortium for PT, represented by eight public educational institutions in the state of São Paulo, made it possible to apply PT in a unified manner in 2019, 2021 and 2022.

The PT of São Paulo Public Schools of Nursing is a cognitive assessment, without selection or classification, applied to undergraduate nursing students at public universities in the state of São Paulo, proposed with the aim at: assessing nursing students’ cognitive knowledge through objective questions; monitoring students throughout their training process; enabling institutional assessment through performance analysis; strengthening nursing education by identifying training gaps and proposing alternatives for academic management; promoting integration between educational institutions, strengthening the discussion about the quality of nursing education; and enabling students to self-assess the knowledge acquired and monitor their progress throughout the course.

Given the need to seek to achieve quality in nursing education, PT can be used as a teaching-learning strategy complementary to the pedagogical project, providing cognitive assessment of pertinent and expected content in nursing training. Hence, PT constitutes a longitudinal, intra-, and inter-institutional assessment process, based on constructivist theory, aiming to overcome the limitations of traditional learning assessment^(1,8)^.

There is evidence that PT provides benefits for students, professors and academic management^(9)^. However, in order to obtain better and more reliable results, it is important that the test is consistent with the nurse profile expected to be trained, valid in terms of its ability to assess what is expected from this student and training, based on the creation of spaces for discussion of themes and issues.

When carried out collaboratively, with different institutions, PT represents a benchmarking opportunity, with the possibility of comparing the effectiveness of curricular approaches and assessing possible changes in course curricula^(10)^. In this context, the establishment of consortia is an important strategy for obtaining gains in the PT preparation and application process.

In medicine, the first Brazilian consortium for PT was implemented around 15 years ago^(11)^ and, from 2013, the Brazilian Association of Medical Education (ABEM - *Associação Brasileira de Educação Médica*) has encouraged the carrying out of PT and the establishment of institutional consortia, being that, currently, 18 consortia are created, in which more than 220 Brazilian medical schools participate^(12)^.

No reports were identified on the national and international scene about PT application in undergraduate nursing courses and/or about the establishment of consortia for this purpose. Therefore, this study aims to report the experience of implementing the São Paulo Nursing Courses Consortium for PT.

## METHOD

### Study Design

This is an experience report, referring to the work of the São Paulo Nursing Courses Consortium for PT in applying tests to undergraduate nursing students in 2019, 2021 and 2022.

### Study Setting and Protocol

The São Paulo Nursing Courses Consortium for PT is made up of eight public educational institutions in the state of São Paulo, namely: EERP-USP; *Escola de Enfermagem da USP* (EEUSP); EPE-UNIFESP; *Faculdade de Medicina de Marília* (FAMEMA); *Faculdade de Medicina de São José do Rio Preto* (FAMERP); *Universidade Estadual Paulista Faculdade de Medicina de Botucatu* (UNESP); UNICAMP School of Nursing; and *Universidade Federal de São Carlos* (UFSCar).

This study uses secondary data, originating from the consortium’s work records and the database resulting from PT application in 2019, 2021 and 2022, with data collection carried out in March 2023. It is worth highlighting that, in 2019, testing took place in person at the facilities of each of the participating institutions; due to the pandemic, the test was not applied in 2020 and was administered remotely in 2021 and 2022. For all editions, the consortium had the operational support of a specialized company, responsible for applying and analyzing the test results.

The tests were prepared by professors from the participating institutions, structured in 120 objective questions, distributed in six areas of knowledge, selected based on the current Undergraduate Nursing Course Brazilian National Curriculum Guidelines recommendations^(13)^: collective health (n = 20); management (n = 15); child and adolescent health (n = 20); women’s health (n = 20); adult and elderly health (n = 35); and mental health (n = 10). Among the 120 questions, 35 pre-tested questions are used, which are previously applied questions distributed evenly across the six areas of knowledge, that presented excellent or good psychometric performance, answered correctly by a group of students with the expected skill level and with little correct answers by students with an immediately lower skill^(14)^.

It was agreed to use four-item questions, one correct and three incorrect (distractors). All questions present justification for the correct item, comments for distractors and references. Students’ total score is calculated by the number of correct questions. In the test, there is no “do not know” option, nor penalties for selecting incorrect options. The test is analyzed globally using item response theory, with a process of psychometric analysis of items and analysis of the difficulty and discrimination indices of the questions.

The degree of difficulty of each question is categorized into “very easy”, “easy”, “medium”, “difficult” and “very difficult”, and the breakdown into “deficient item, must be rejected”, “deficient item, subject re-elaboration”, “good, but subject to improvement” and “good”.

Through descriptive analysis, this experience report presents the work process and progress made by the consortium in applying PT in 2019, 2021 and 2022, which saw, respectively, the participation of 1,105, 1,138 and 1,175 students, respectively.

### Ethical Aspects

This study followed ethical standards and recommendations in accordance with Brazilian National Health Council (CNS – *Conselho Nacional de Saúde*) Resolution 466/12. All participating institutions registered their consent to use the database, results, and performance in PT for analysis and scientific dissemination purposes. Due to the use of a secondary database, Informed Consent Form was waived. The data was anonymized, making it impossible to identify students and/or educational institutions during data processing and analysis. The study was submitted for analysis by a Research Ethics Committee, and approved, in January 2023, under Opinion 5,865,717.

## RESULTS

The consortium’s activities are led by a working group composed of two faculty members representatives from each of the eight participating institutions, under the coordination of two professors, responsible for managing the test and liaising with the working group and institutions. This working group is responsible for managing the test development and operationalization.

The work process involves the active participation and collaboration of the consortium working group, in addition to the participation of expert professors in the areas of knowledge, representatives of the participating institutions, who share knowledge, experiences and resources.

Annually, each institution nominates two representatives to act as consortium members. Among these nominees, the coordinator and vice-coordinator and coordinators for each of the six areas are chosen with peer approval. Additionally, representatives from each educational institution nominate professors from their institution to form the panel of experts by area, thus guaranteeing the participation of colleagues from all institutions in all areas.

The consortium’s activities are carried out through in-person and virtual meetings, as described in Chart 1, and begin with the definition of learning objectives, which guide the establishment of axes and themes for each of the areas of knowledge.

**Chart 1:** Synthesis of activities to carry out the progress test of public schools of nursing in são paulo – Ribeirão Preto, SP, Brazil, 2024.

Step	Activity description	Responsible bodies
Planning	Definition of work schedule, including definition of test application date; formation of a working group with representation from the eight educational institutions; definition of coordinators for knowledge areas.	Consortium
Theme review	Review and selection of axes and themes by area of knowledge through internal discussion between areas with the participation of area coordinators and experts from the institutions.	Area coordinators
Question distribution and request	Validation of selected themes and distribution of each theme to four institutions.	Consortium
Professor training	Holding a training workshop for professors on good practices for preparing objective questions.	Consortium
Question preparation	Preparation of questions by institutions, including internal review stage.	Educational institutions
Question reception	Reception, formatting review, anonymization, and coding of questions.	Consortium coordinators
Question selection	Discussion and selection of questions by area, with the participation of expert professors in the area from all institutions.	Area coordinators
Question validation	Review of selected questions for the final test composition.	Consortium
Student registration	Test publicity, information sessions and voluntary student registration.	Consortium, institutions, and students
Test application	Registration validation by students and teste application.	Students and contractor
Analysis and dissemination of results	Analysis of results by the contracted company, with performance disseminated to students, institutions, and consortium.	Contracted company, consortium, institutions, and students

The 120 test questions, distributed across the six areas of knowledge, seek to cover the main topics and skills that undergraduate nursing students must develop during the course, carrying out a cognitive assessment of important topics for nurses’ professional performance. Content is defined and reviewed by experts in each area, and questions are prepared by professors from all participating institutions.

In the preparation process, pre-tested question themes are not considered in the distribution between the institutions, with 85 themes being distributed among the eight institutions. All institutions prepare questions in the six areas of knowledge. Four questions per topic are expected, prepared by four different institutions, totaling 340 questions for review and selection of 85 questions that, together with the 35 pre-tested questions, make up the 120-question test to be applied.

Aiming at standardizing the structure of questions as well as qualifying the preparation of questions, a training workshop is held annually for professors on good practices for preparing objective questions (Chart 2).

**Chart 2:** Synthesis of good practices for preparing questions for the progress test of public schools of nursing in São Paulo – Ribeirão Preto, SP, Brazil, 2024.

Each question must: – Focus on a concept relevant to nurses’ role; – Preferably assess knowledge application or data interpretation, avoiding questions that only require memorization of content; – Have stated a clear question that can be answered without the help of alternatives; – Have a relatively long statement, but not excessively, in order to contextualize what is intended; – End the statement with a question, indicating the focus of what is being assessed; – Have four alternatives, only one of which is correct; – Have plausible alternatives, which present only necessary information; – Have alternatives of homogeneous size between them. Must be avoided: – Create true or false questions; – Create statements that end with the question “Which of the following alternatives is correct?”; – Formulate questions in a negative way, e.g., “all of the following statements are correct, except”, “the incorrect one is” or “which of the alternatives is NOT true/correct” – Alternatives with different groupings to choose the correct sequence set (e.g., VI, II, III and I); – Use exhaustive or imprecise terms, such as always, never, generally, frequently; – Use excessive and unnecessary information that confuses students; – Use information that presents elements that help or induce correct answers even without knowing what is being asked.

Each institution must prepare, on average, 43 questions on previously defined topics in different areas of knowledge, with an average deadline of 60 days.

The questions are prepared and reviewed internally at each institution, in addition to being anonymized, organized by area and theme and coded, without the possibility of identifying the institution from which the question originated during the review and selection process. The questions are reviewed and validated through discussion and consensus between consortium members and experts in the six areas of the test and, subsequently, reviewed and validated by consortium members to compose the final test to be applied to students.

The participatory process, involving expert professors from all institutions, allows for a more comprehensive coverage of the curriculum and topics relevant to nursing training. Conducting the process by representatives from different institutions and perspectives reduces possible test bias. Additionally, a high number of questions analyzed favors the composition of a broad question bank, which allows review and selection of questions with greater alignment with the intended assessment.

Once test validation is completed, it is applied on a date previously defined during the planning process, together with the establishment of the annual schedule so that institutions can include it in their academic schedule and then, allow broad student participation. The test is administered annually, is voluntary and does not involve costs for students.

Test formatting, layout, and printing (for 2019) or virtual environment setting (for 2022 and 2023), correction of answers, analysis, and dissemination of results to students and schools and issuance of participation certificates to students are centralized, carried out by a company hired by schools with experience in applying tests of this type.

The consortium is responsible for disseminating and promoting guidance and awareness-raising actions for students, in a shared manner with institutions, which internally carry out dissemination and guidance for students.

Each institution registers its students and sends the list of registrants to the contracted company for registration in the system and operational procedures for applying the test, analyzing, and publishing the results. All students regularly enrolled in one of the eight institutions participating in the consortium are eligible to participate in PT. After the registration period, students validated their registration with the company in the week before taking the test.

The test was carried out simultaneously for all participants from all institutions, administered in person in 2019 and virtually in 2021 and 2022. In both models, the test had the same composition, 120 questions, and took four hours to complete.

After taking the test, students have access to the commented answer sheet with references and analysis of their performance, individually, comparing their performance with that of students in the same year of course at their institution and the institutions in the consortium. If students have taken the test in previous editions, it is possible to view their progression between tests.

From an institutional point of view, each school received an analysis of its students’ performance, presented by year of course and by area of knowledge, with a global comparison with the institution with the highest or lowest performance, without identifying institutions. Internally, each institution is responsible for analyzing its results and using the information in the most appropriate way, both from the point of view of feedback to professors responsible for the content covered and from the point of view of curriculum review and strategies related to the pedagogical project.

In the analysis of the tests applied, a progressive increase in the absolute and proportional number of students was observed, with a participation of 46.1% of the total number of students enrolled in the eight institutions in 2019 and 57.3% in 2022 (Table 1). Despite the greater participation, actions to raise awareness and encourage student participation must be continually promoted aiming for the participation of as many students as possible and an even more reliable overview of the assessment carried out.

**Table 1:** Student participation in the Progress Test – Ribeirão Preto, SP, Brazil, 2024.

Year	Enrolled students	Participating students	Percentage
2019	2,399	1,105	46.1%
2021	2,076	1,138	54.8%
2022	2,050	1,175	57.3%

In difficulty index analysis (Table 2), a disproportionate distribution between “easy” and “very easy” questions and “difficult” or “very difficult” questions is observed, with a predominance of those of medium difficulty.

**Table 2:** Difficulty index categorized by year of application of the Progress Test in Public Schools of Nursing in São Paulo – Ribeirão Preto, SP, Brazil, 2024.

Difficulty	2019	2021	2022
Very easy	12	17	22
Easy	39	34	35
Medium	61	67	60
Difficult	7	2	2
Very difficult	1	0	1

Concerning discrimination index, there was a gradual increase in questions classified as “good” or “good, but subject to improvement”, which together represented 68.3%, 80.0% and 82.5%, respectively, of the total number of test questions applied in 2019, 2021 and 2022 (Table 3), indicating a constant process of improvement in question formulation.

**Table 3:** Discrimination index categorized year of application of the Progress Test in Public Schools of Nursing in São Paulo – Ribeirão Preto, SP, Brazil, 2024.

Discrimination	2019	2021	2022
Deficient item, must be rejected	16	6	8
Deficient item, subject re-elaboration	22	18	13
Good, but subject to improvement	33	33	19
Good	49	63	80

## DISCUSSION

The present study made it possible to report the experience of the consortium of eight public schools in São Paulo in applying PT as well as presenting a descriptive analysis of the work process and the results obtained in the test, considering the participation of students and the classification of difficulty indices and discrimination of questions.

The experience of working in a consortium has allowed cognitive assessment of student learning to be carried out with better results and higher quality. Working with different institutions avoids endogeny and adds different perspectives to the process, allowing for a more comprehensive assessment of students and advancing in terms of clarity of what is measured, test rigor and quality.

PT, through its objective questions, makes it possible to present the student with situations, problems and conditions similar to those observed in professional practice^(15)^. However, ensuring quality assessment is essential.

To this end, promoting professor qualification strategies and the use of good practices in preparing objective questions leads to the availability of questions capable of assessing more complex problems, increasing test validity and reliability^(16)^. Holding workshops to prepare objective questions, presented in this report, is a strategy also successfully adopted in medical schools^(17)^. Additionally, for professors who author questions, training, peer review, monitoring, and communication of expectations by more experienced professors, and feedback on the structure of questions and their psychometric performance are considered good practices^(18,19)^.

In addition to developing teaching capacity in preparing questions and improving question psychometric quality, workshops increase the interest and involvement of professors with the test^(20)^, also allowing the exchange of experiences and information about the content covered in each course.

PT question quality indicates the progressive improvement of the questions prepared by the consortium. Even though most questions have good technical quality, it is possible to identify opportunities for improvement in the process of preparing questions, serving as a basis for future professor training actions. It should be noted that the analysis of the psychometric indices of the questions is fundamental, since faulty questions lead to a systematic error in student performance assessment, compromising the validity and interpretation of test results and penalizing students^(18,21)^.

From the perspective of commitment and collaboration, the establishment and operations of the consortium have allowed the exchange of knowledge and joint action of different actors. Working collaboratively, with representatives from different institutions, allows to understand and use the content of different areas of knowledge from different perspectives and approaches, enriching the test structure and quality. Implementing the consortium also enables a collaboration process that goes beyond PT, favoring the exchange of information and experiences between institutions^(1)^.

From an operational perspective, working in a consortium consolidates collective and interinstitutional management and scales the process, favoring its efficiency and optimizing costs. Even though it involves a high number of participating students, there are operational gains in terms of automated correction and communication of answer sheets and performance feedback^(15)^.

It is worth highlighting that institutional support, including commitment and involvement of directors, coordinators, and professors, is essential both in test preparation and student participation and in analysis and discussion of results^(17)^. For the potential use of PT as an academic management tool, it is necessary for institutions to take an active role in the analysis and contextualization of results, allowing managers to make inferences and decisions about content and relevant pedagogical strategies^(22)^.

When applied jointly by different institutions, it is observed that participation in PT is related to the way in which the institution is involved in the test, and student participation is higher in institutions that are more actively involved, recognize assessment as an important part of the curriculum and visualize the possibilities of following the teaching-learning process. In these institutions, students are more encouraged to understand the principle of the test and use the results as feedback on their knowledge progress; consequently, they feel more motivated to participate and report less fear about taking the test^(23)^.

Therefore, incorporating the test into assessment strategies should be sought by institutions through the optimization of the experience that the test provides to students, including its understanding, contextualization and adequate feedback^(24)^.

PT represents an innovation in nursing education, with potential application in the assessment of students’ cognitive performance in a manner aligned with the professional skills expected of nurses, favoring the development of skills and preparation to deal with the challenges of professional practice.

Based on the experience of other areas of activity and application contexts, it is understood that the report presented here advances knowledge about student knowledge assessment strategies and can even be a basis for applying tests in other contexts, such as residency or professional licensing tests.

Finally, it is worth highlighting, as a limitation, the incipient use of results as an academic management tool. It is understood that the test has been efficient from the point of view of global institutional analysis of student performance, and for feedback and self-management of knowledge by students; however, actions and proposals for changes in the pedagogical project based on PT results are still incipient.

## FINAL CONSIDERATIONS

The establishment of the São Paulo Nursing Courses Consortium for PT has allowed the test to be applied interinstitutionally, expanding the test scope and quality, thus seeking better accuracy in assessing nursing students’ cognitive performance.

Increased student participation and greater involvement of institutions in the process of preparing the test and using its results for academic management are consequences of a continuous process of awareness, recognition, and credibility on the part of both students and professors.

Although student participation has increased year after year, it is expected to increase in the next test offerings, aiming to have a more accurate overview of the assessment of the cognitive performance of all nursing students.

In general, it is observed that the questions prepared by the consortium have undergone a gradual increase in quality resulting from improvement actions implemented by the consortium, which have acted with greater maturity and clarity in PT processes and implications. Despite this, it should be noted that, for future applications, test development must consider elaborating questions of different levels of difficulty, with a greater proportion of questions of greater difficulty.

Finally, through this experience report, it is hoped to encourage PT implementation in undergraduate nursing courses in other contexts, whether at institutional or interinstitutional level, always with a view to improving nursing training quality.
